# Author Correction: Sema7A/PlxnCl signaling triggers activity-dependent olfactory synapse formation

**DOI:** 10.1038/s41467-019-11055-6

**Published:** 2019-07-10

**Authors:** Nobuko Inoue, Hirofumi Nishizumi, Hiromi Naritsuka, Hiroshi Kiyonari, Hitoshi Sakano

**Affiliations:** 1 Department of Brain Function, University of Fukui School of Medicine, 23-3 Shimo-aizuki, Matsuoka, Fukui, 910-1193 Japan; 2 Department of Physiology, Graduate School of Medicine, The University of Tokyo, 7-3-1 Hongo, Bunkyo-ku, Tokyo, 113-0033 Japan; 3 RIKEN Institute, 2-2-3 Minatojima-minamimachi, Chuo-ku, Kobe, 650-0047 Japan

**Keywords:** Axon and dendritic guidance, Olfactory bulb

Correction to: *Nature Communications* 10.1038/s41467-018-04239-z, published online 9 May 2018.

The original version of this Article contained errors in Figs. 4 and 7. In Fig. 4c, the representative image of anti-GluR1-labelled PlxnC1 conditional knockout glomeruli was inadvertently replaced with a lower magnification duplicate of the equivalent image in Fig. 4a. The correct version of Fig. 4 is:

which replaces the previous incorrect version which is:

In Fig. 7b, the representative image of myc-PlxnC1/FLAG-SAP90 expressing cells treated with Sema7A Y213S was inadvertently replaced with an inverted duplicate of the image directly above, showing cells without Sema7A treatment. The correct version of Fig. 7 is:

which replaces the previous incorrect version which is:

This has been corrected in both the PDF and HTML versions of the Article. Additional raw data that support the findings of these experiments can be found as Supplementary Information associated with this Correction.

