# Supplementary material for: Sema7A/PlxnCl signaling triggers activity-dependent olfactory synapse formation
Source: Nat Commun. 2018 May 9;9:1842. doi: 10.1038/s41467-018-04239-z (PMC5943276; doi:10.1038/s41467-018-04239-z)
Supplement: Supplementary file 1 — Supplementary Information [file 41467_2018_4239_MOESM1_ESM.pdf]

Glomerular formation and localization in the OB

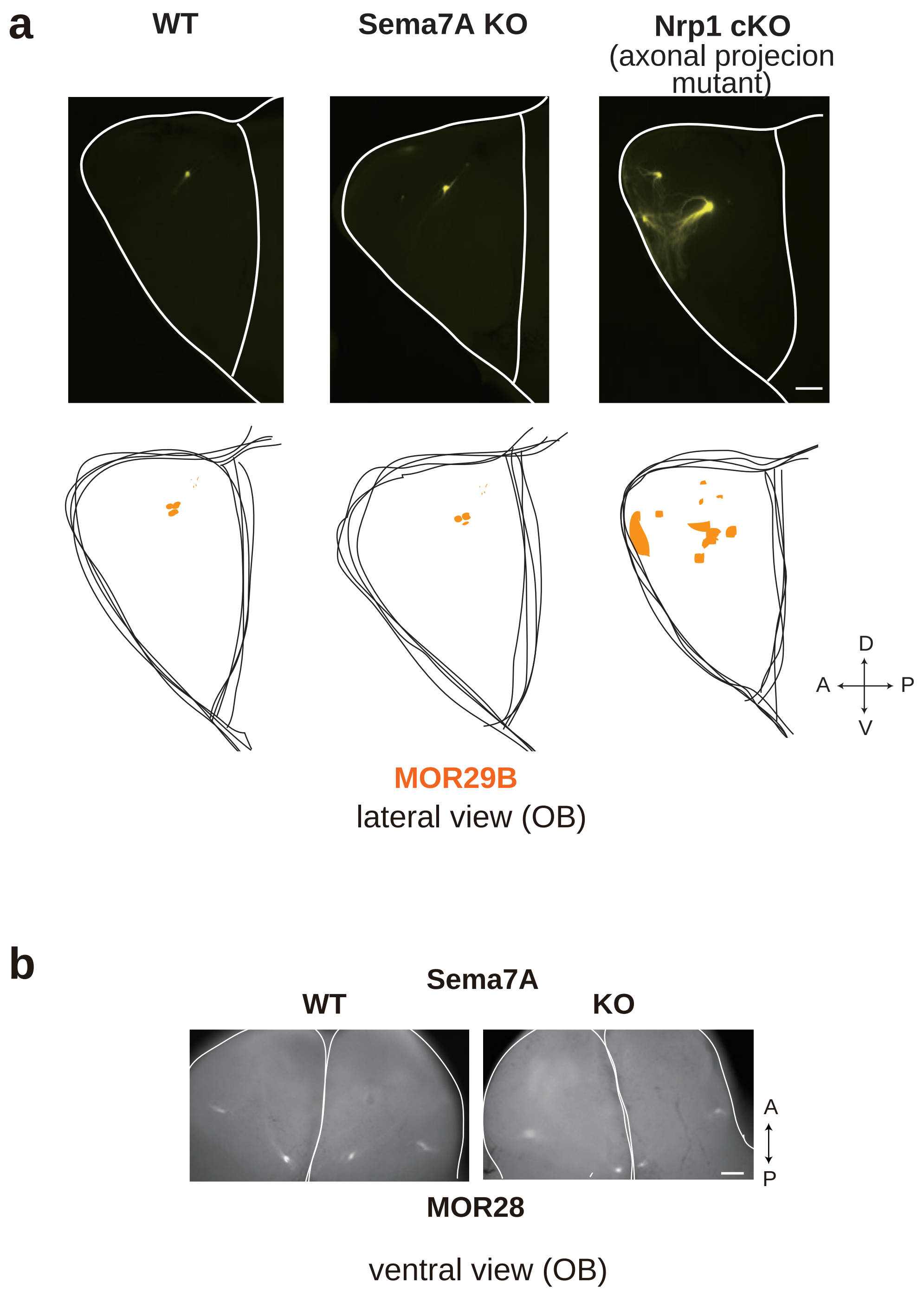

Supplementary figure 1   Inoue et al.

Supplementary figure. 1. Targeting of OSN axons in the Sema7A KO. a, The tg *MOR29B* gene was tagged with *EYFP* for fluorescent staining of the MOR29B glomeruli. Lateral views of the OB are shown. Tg animals were mated with Sema7A total KO or OSN-specific cKO of Nrp1 and analyzed at P20. Relative locations of the MOR29B glomeruli are schematically shown. n=4 for each mouse strain. Scale bar, 50 μm. **b**, The *MOR28* gene was tagged with *EGFP* for fluorescent staining of the MOR28 glomeruli. The MOR28 KI mice were crossed with Sema7A KO. Ventral views of the OB are shown. The WT and Sema7A total KO were analyzed at P10. n=5 for each mouse strain. Scale bar, 50 μm. D, dorsal; V, ventral; A, anterior; and P, posterior.

M/T-cell specific PlxnC1 cKO

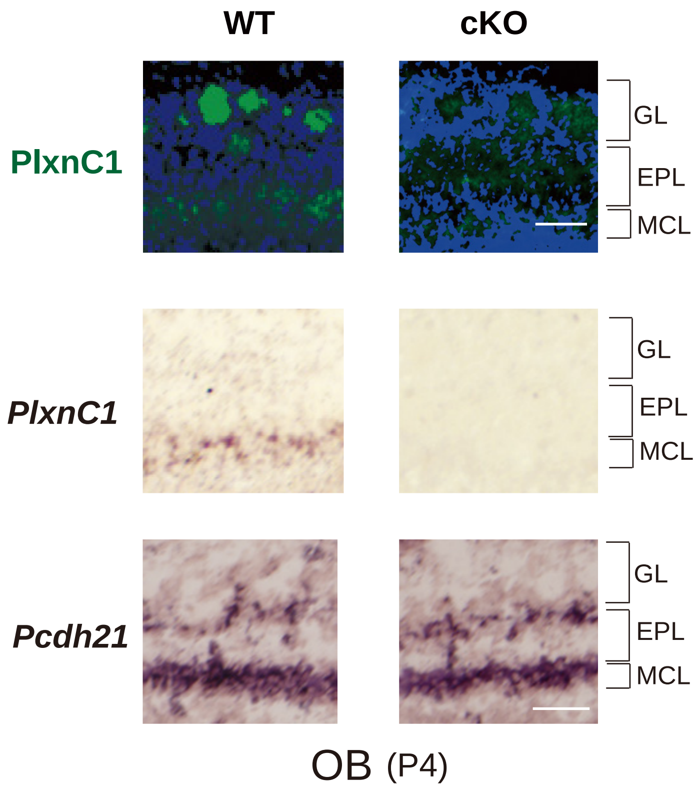

Supplementary figure 2   Inoue et al.

Supplementary figure 2. M/T cell-specific PlxnC1 cKO. Absence of PlxnCl expression in the cKO. OB sections at P4 were immunostained with antibodies against PlxnC1 for both the WT and cKO. Transcription of the *PlxnC1* gene was also analyzed by *in situ* hybridization. *Pcdh21*, M/T-cell marker. Scale bars, 50 μm. GL, glomerular layer; EPL, external plexiform layer; and MCL, mitral cell layer.

Sema7A expression

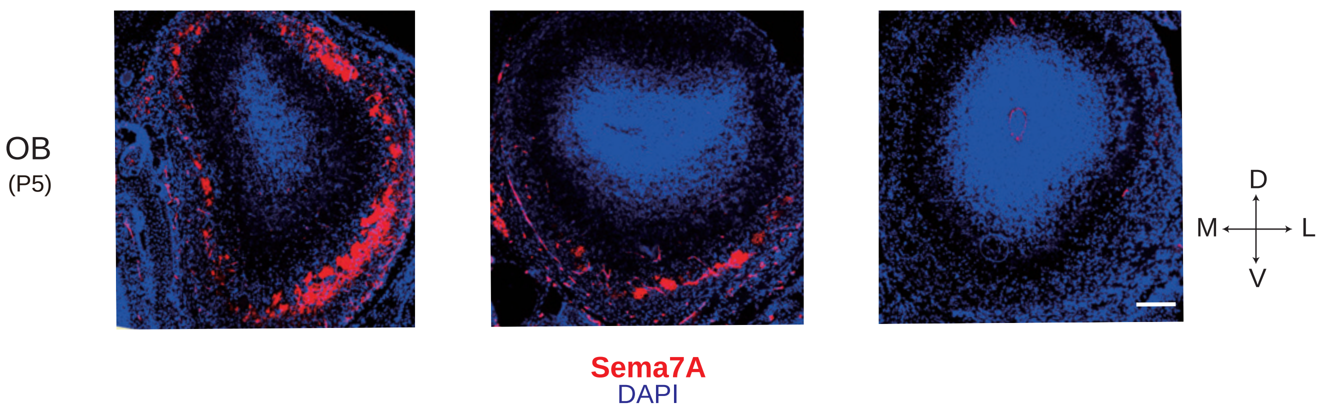

Supplementary figure 3 Inoue et al.

Supplementary figure 3. Detection of Sema7A in the OB. OB sections of the WT, CNG-A2 KO, and Sema7A total KO at P5 were immunostained with anti-Sema7A antibodies (red) and counterstained with DAPI (blue). Scale bar, 10 μm. D, dorsal; V, ventral; M, medial; and L, lateral.

Glomerular formation and localization in the OB

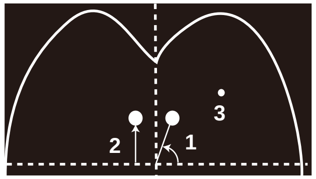

MOR29B glomeruli	WT at P6 (n=8)	Sema7A KO at P6 (n=8)	Nrp1 OSN-specific KO at P6 (n=4)
Dorsal view (OB size) (mm^2)	0.1325 ± 0.010	0.1325 ± 0.0095 (n.s.)	0.1281 ± 0.007 (n.s.)
**1.** Radial position of medial glomeruli (in degrees where 90° is the ventral midline)	75.94 ± 2.33	78.40 ± 1.55 (n.s.)	ec 65.71 ± 4.890 **
**2.** Distance of medial glomeruli from the caudal end of the OB (μm)	201.25 ± 13.94	197.5 ± 14.40 (n.s.)	ec 345.71 ± 46.50 **
**3.** The number of ectopic glomeruli	0.5 ± 0.15	0.5 ± 0.12 (n.s.)	2.1 ± 0.34 **
**MOR28 glomeruli**	**WT at P8 (n=5)**	**Sema7A KO at P8 (n=6)**	**Sema3F OSN-specific KO at P8 (n=4)**
Ventral view (OB size) (mm^2)	0.17 ± 0.0209	0.172 ± 0.0146 (n.s.)	0.165 ± 0.008 (n.s.)
**1.** Radial position of medial glomeruli (in degrees where 90° is the ventral midline)	76.4 ± 3.611	76.2 ± 3.429 (n.s.)	ec 43.42 ± 3.579 **
**2.** Distance of medial glomeruli from the caudal end of the OB (μm)	145.4 ± 28.72	146.8 ± 29.60 (n.s.)	ec 174.57 ± 16.69 **
**3.** The number of ectopic glomeruli	0.4 ± 0.48	0.4 ± 0.5 (n.s.)	2.2 ± 0.45 **

means ± SE. **p<0.01; n.s.:no significant difference was found.

EYFP-tagged MOR29B glomeruli at P6 and MOR28 at P8 were analyzed in the WT, Sema7A KO, OSN-specific Nrp1 KO, and OSN-specific Sema3F KO. The OB size (mm^2), radial position (degree) and distance from the end of OB (μm) were measured. **p<0.01 (Students t test). No significant difference in glomerular formation was found between the WT and Sema7A KO. A schematic diagram for the glomerular measurements is shown. ec, ectopic glomeruli.

Supplementary table 1 Inoue et al.
